# Application of Liquid-Liquid Extraction for N-terminal Myristoylation Proteomics

**DOI:** 10.1016/j.mcpro.2023.100677

**Published:** 2023-11-09

**Authors:** Kazuya Tsumagari, Yosuke Isobe, Yasushi Ishihama, Jun Seita, Makoto Arita, Koshi Imami

**Affiliations:** 1 Proteome Homeostasis Research Unit, RIKEN Center for Integrative Medical Sciences, Yokohama, Kanagawa, Japan; 2 Laboratory for Metabolomics, RIKEN Center for Integrative Medical Sciences, Yokohama, Kanagawa, Japan; 3 Laboratory for Integrative Genomics, RIKEN Center for Integrative Medical Sciences, Yokohama, Kanagawa, Japan; 4 Division of Physiological Chemistry and Metabolism, Graduate School of Pharmaceutical Sciences, Keio University, Tokyo, Japan; 5 Cellular and Molecular Epigenetics Laboratory, Graduate School of Medical Life Science, Yokohama City University, Yokohama, Kanagawa, Japan; 6 Department of Molecular Systems Bioanalysis, Graduate School of Pharmaceutical Sciences, Kyoto University, Kyoto, Japan; 7 Laboratory of Clinical and Analytical Chemistry, National Institute of Biomedical Innovation, Health and Nutrition, Osaka, Ibaraki, Japan; 8 Human Biology-Microbiome-Quantum Research Center (WPI-Bio2Q), Keio University, Tokyo, Japan

**Keywords:** myristoylation, lipidation, liquid-liquid extraction, peptide enrichment, mass spectrometry, post-translational modification, co-translational modification

## Abstract

Proteins can be modified by lipids in various ways, for example, by myristoylation, palmitoylation, farnesylation, and geranylgeranylation—these processes are collectively referred to as lipidation. Current chemical proteomics using alkyne lipids has enabled the identification of lipidated protein candidates but does not identify endogenous lipidation sites and is not readily applicable to *in vivo* systems. Here, we introduce a proteomic methodology for global analysis of endogenous protein N-terminal myristoylation sites that combines liquid-liquid extraction of hydrophobic lipidated peptides with liquid chromatography-tandem mass spectrometry using a gradient program of acetonitrile in the high concentration range. We applied this method to explore myristoylation sites in HeLa cells and identified a total of 75 protein N-terminal myristoylation sites, which is more than the number of high-confidence myristoylated proteins identified by myristic acid analog-based chemical proteomics. Isolation of myristoylated peptides from HeLa digests prepared with different proteases enabled the identification of different myristoylated sites, extending the coverage of *N*-myristoylome. Finally, we analyzed *in vivo* myristoylation sites in mouse tissues and found that the lipidation profile is tissue-specific. This simple method (not requiring chemical labeling or affinity purification) should be a promising tool for global profiling of protein N-terminal myristoylation.

## Highlights

• Isolation of myristoylated peptides by liquid-liquid extraction.
• Identification of intact myristoylated peptides without the need for chemical probes.
• Use of multiple digestive enzymes to expand coverage of myristoylation sites.
• *In vivo* myristoylation profiling of individual mouse organs.

## Graphical Abstract

**Figure:**
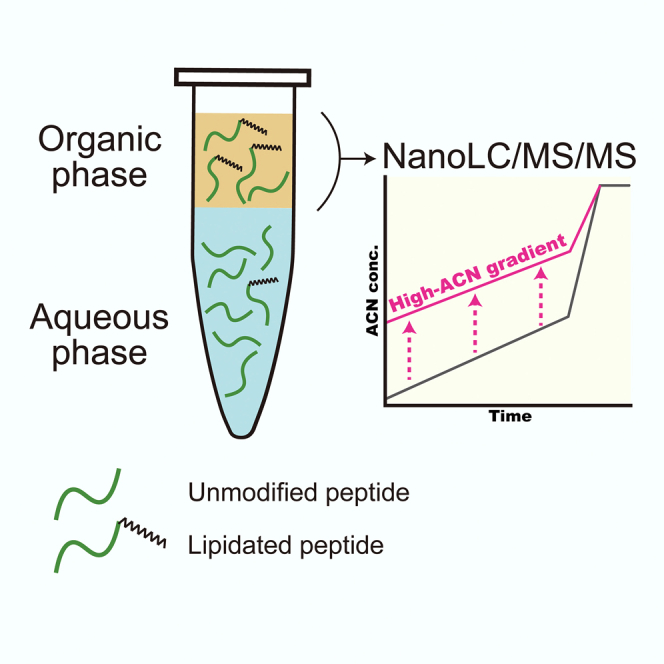

## In Brief

Protein N-terminal modification by myristic acid, termed myristoylation, is functionally important. We present a method to directly identify myristoylation sites on a proteomic scale by protein digestion followed by liquid-liquid extraction of hydrophobic myristoylated peptides into organic phases. The method was employed to profile myristoylation in various mouse organs.

Numerous proteins are covalently modified with a variety of lipid molecules, as exemplified by myristoylation of protein N-terminal glycine and palmitoylation, farnesylation, and geranylgeranylation of cysteine. These processes are collectively referred to as lipidation (1, 2). Lipidation alters protein hydrophobicity, thereby affecting stability, localization, and interaction with other proteins (1, 2). Especially, protein N-terminal myristoylation is a ubiquitous protein modification catalyzed by *N*-myristoyltransferases (NMTs) and has been implicated in the development and progression of diseases. For instance, myristoylation of proto-oncogene tyrosine-protein kinase SRC influences protein folding and promotes a switch to the active conformation (3). Elevated myristoylation levels of SRC can cause cancer (3), suggesting that lipidation may be involved in disease mechanisms. While protein lipidation, including myristoylation, is one of the key modifications that drive many aspects of biological processes, it has been less studied than other modifications such as phosphorylation, possibly due to the technical difficulties in analyzing low-abundance hydrophobic peptides as well as the structural diversity of lipid modifications.

Proteomics using nanoscale liquid chromatography/tandem mass spectrometry (nanoLC/MS/MS) is a powerful approach, particularly in studies of post- or co-translational modifications of proteins. In most cases, myristoylated proteins have been analyzed using a chemical proteomic approach, in which proteins are metabolically labeled with lipid-mimicking chemical probes and isolated by affinity purification *via* click chemistry, followed by identification of unmodified peptides from digested myristoylated proteins (4). For instance, Thinon *et al.* (5) performed protein N-terminal myristoylome analysis of HeLa cells using clickable myristic acid analogs, and identified 70 co-translationally myristoylated proteins with high confidence. While this chemical proteomic approach is effective in profiling the lipidated proteome, it has several limitations. First, site-level information is often lost, since probe-tagged peptides are not eluted after digestion. To solve this problem, Broncel *et al.* (6) developed a multifunctional probe that enables direct identification of probe-tagged peptides, detecting a total of 81 tagged peptides in three human cell lines. Second, chemical probes may not capture endogenous lipid modifications due to structural differences from endogenous lipids. Also, chemical probe-based metabolic labeling can cause off-target effects; metabolic labeling with a myristic acid analog is not entirely specific for myristoylation as it also involves other targets, mainly GPI-anchored proteins, as well as palmitoylated proteins which may incorporate the myristic acid analog, and some unidentified proteins (7). Hence, additional evidence that affinity capture of myristoylated proteins is indeed inhibited by a specific NMT inhibitor is typically required to confirm *bona fide* targets (5, 7). Third, the metabolic labeling approach is not readily applicable *in vivo*, such as in human tissues. Thus, a new strategy that overcomes these limitations is needed.

To directly identify modification sites of interest, modified peptides have to be biochemically enriched from protein digests using an affinity matrix, such as immobilized antibodies, prior to nanoLC/MS/MS, since their abundance is relatively low compared to that of unmodified peptides. Such peptide-level enrichment is the key to pinpointing modification sites with regulatory functions, but enrichment of lipidated peptides is challenging. Until now, a combination of subcellular fractionation, pre-fractionation of proteins or peptides, and separation of hydrophobic lipidated peptides on nanoLC/MS/MS has led to the identification of 72 non-redundant *Arabidopsis thaliana* myristoylated proteins and 69 human myristoylated proteins (7, 8, 9). Liquid-liquid extraction (LLE) is commonly used to separate lipids and nonlipid species (*e.g.*, proteins) based on partitioning of lipids into an organic phase. Indeed, LLE is effective for isolating lipidated peptides (10, 11), but has not been applied to global analysis of myristoylated peptides from complex samples. Herein, we sought to explore the utility of LLE for lipidation proteomics. We show that our developed method can efficiently enrich endogenous myristoylated peptides from biological samples. We provide a proof-of-concept of this approach targeting the HeLa cell myristoylome, and then describe a further application for quantitative analysis of *in vivo* lipidation sites in mouse tissues.

## Experimental Procedures

### Cell Culture

HeLa cells were cultured in Dulbecco's modified Eagle’s medium (FUJIFILM Wako) containing 10% FBS (Thermo Fisher Scientific) and 100 U/ml penicillin and 100 μg/ml streptomycin (FUJIFILM Wako). Cells were harvested, washed with PBS, and stored at −80 °C until use.

### Mouse

Animal experimental procedures were approved by the Animal Care and Use Committee of RIKEN. A male C57BL/6J mouse was purchased from CLEA Japan Inc, bred under specific pathogen-free conditions, and sacrificed at 12 weeks of age. Organs were quickly dissected, snap-frozen with liquid nitrogen, and stored at −80 °C until use.

### Protein Extraction and Digestion

HeLa cells were suspended in 8 M urea buffer including 100 mM Tris-HCl (pH 8.5), 10 mM tris(2-carboxyethyl)phosphine (TCEP; FUJIFILM Wako), 40 mM 2-chloroacetamide (CAA; FUJIFILM Wako), and 28.3 U of benzonase (Merck) and agitated at room temperature for 30 min. Then, the protein was extracted and denatured simultaneously by sonication for 20 min on ice. The protein concentration was determined by means of bicinchoninic acid (BCA) assay (Thermo Fisher Scientific). The protein solution was diluted 5-fold with 50 mM ammonium bicarbonate (ABC; FUJIFILM Wako), and proteins were digested overnight with LysC (FUJIFILM Wako) and trypsin (sequence grade; Promega) at the protein:enzyme ratio of 100:1 for each enzyme at 25 °C. When GluC (FUJIFILM Wako) or chymotrypsin (ROCHE) was used, the protein solution was diluted 10-fold with 50 mM phosphate buffer (pH 8.5; for GluC) or ABC (for chymotrypsin) and digested at the protein:enzyme ratio of 50:1. Digestion was halted by acidifying the mixture with trifluoroacetic acid (TFA; FUJIFILM Wako). The obtained digests were purified on MonoSpin C18 reversed-phase columns (GL Sciences), evaporated using a SpeedVac (Thermo Fisher Scientific), and subjected to LLE as described below.

Mouse organs were suspended in 5% sodium dodecyl sulfate (SDS) buffer containing 100 mM Tris-HCl (pH 8.5), 10 mM TCEP, and 40 mM CAA and crushed using zirconia beads (Tomy) and TissueLyser (Qiagen). Proteins were inactivated at 95 °C for 5 min, extracted by sonication for 20 min on ice, and purified by methanol-chloroform precipitation. The protein concentration was determined using BCA assay. Proteins were digested with trypsin and LysC and the peptides were purified using MonoSpin C18 columns, evaporated, and subjected to LLE.

### Liquid-Liquid Extraction

All organic solvents used for LLE were obtained from FUJIFILM Wako. In most experiments, 100 μg of desalted protein digest was subjected to LLE. In the experiment comparing solvents (related to Fig. 2), 50 μg of digest was subjected to LLE. Digests were dissolved in 50 μl of 0.5% TFA and vigorously mixed with 65 μl of organic solvent for 10 min using a vortex mixer at room temperature. Following centrifugation at 18,000*g* for 5 min at room temperature, 50 μl of the upper phase (organic phase) was obtained and evaporated using a SpeedVac (Thermo Fisher Scientific).

### NanoLC/MS/MS

A nanoLC/MS/MS system comprising an EASY-nLC 1200 (Thermo Fisher Scientific) and an Orbitrap Eclipse mass spectrometer (Thermo Fisher Scientific) was employed. The mobile phases consisted of (A) 0.1% formic acid and (B) 0.1% formic acid and 80% acetonitrile (ACN). In-house packed columns were prepared as follows: emitters were generated by pulling a 25 cm fused-silica capillary (100 μm inner diameter; GL Sciences) using the P-2000 laser puller (Sutter Instrument). Then, ReproSil-Pur C18-AQ (1.9 μm, Dr Maisch) was packed into the emitter using an air-pressure pump connected to an N_2_ bomb, generating a 15 to 18 cm column (12). Lipidated peptides were separated as follows: first, contaminating hydrophilic peptides were washed out with 38% B for 10 min at the flow rate of 800 nl/min, and then remaining hydrophobic peptides were separated by applying a linear gradient for 43 min (38–70% B over 30 min, 70–99% B over 3 min, and 99% B for 10 min) at the flow rate of 500 (solvent comparison) or 300 nl/min (HeLa cells and mouse organs). MS scanning was initiated 10 min after the start of LC. All spectra were obtained using the Orbitrap analyzer. MS1 scans were performed in the range of 375 to 1500 *m/z* (resolution = 120,000, maximum injection time = “Auto”, and automatic gain control = “Standard”). For the subsequent MS/MS analysis, precursor ions were selected and isolated in a top-speed mode (cycle time = 3 s and isolation window = 1.6 *m/z*) and activated by higher-energy collisional dissociation (HCD; normalized collision energy = 28). Column temperature was set to 50 °C. In the analyses of HeLa cells and mouse organs, FAIMSpro (Thermo Fisher Scientific) was employed, and the samples were analyzed in four runs with different compensation voltages (CVs) (−30/−70; −40/−80; −50/−90; and −60/−100). In the measurements of mouse tissue whole proteomes, peptides were separated by a linear gradient for 90 min (5–10% B over 5 min, 10–36% B over 70 min, 36–99% B over 5 min, and 99% B for 10 min) at the flow rate of 350 nl/min. MS scanning was performed in the data-independent acquisition (DIA) mode. MS1 scans were performed in the range of 350 to 1000 *m/z* (resolution = 120,000, maximum injection time = 45 ms, and automatic gain control = 300%). In the following MS/MS scans, the precursor range was set to 500 to 740 *m/z*, and 60 scans were acquired with the isolation window of 4 *m/z*, with HCD normalized collision energy of 27 (resolution = 15,000, injection time = 22 ms, auto gain control = 1000%, first mass = 120 *m/z*). FAIMS CV was fixed to −45.

In the experiment comparing retention times of synthetic lipidated peptides with HeLa digest (related to supplemental Fig. S1), 200 ng of HeLa digest spiked with 2 pmol synthetic peptides was injected. In the experiment comparing organic solvents (related to Fig. 2), isolated peptides were dissolved in 10 μl of loading buffer consisting of 4% ACN and 0.5% TFA, and an aliquot of 5 μl was injected into the MS. In the HeLa cells and mouse organ profiling (related to Figs. 3 and 4), isolated peptides from 100 μg digest were dissolved in 22 μl of the loading buffer, and a 5 μl aliquot was injected per measurement. In the measurements of mouse organ whole proteomes, 200 ng of digest was injected.

### Data Processing

In most experiments, LC/MS/MS raw data were processed using FragPipe (v.19.0) with the MSFragger search engine (v.3.7), Philosopher (v.4.6.0), and IonQuant (v.1.8.10) (13). Database search was implemented against the UniprotKB/SwissProt (April 2022) human (20,425 sequences) or mouse (17,223 sequences) database with commonly observed contaminant proteins. Cysteine carbamidomethylation as a fixed modification, and methionine oxidation and acetylation on the protein N-terminus as variable modifications were set in all searches. For the identification of myristoylation and palmitoylation sites, myristoylation of protein N-terminal glycine (+210.19836 Da) and palmitoylation of cysteine (+181.2082 Da) were considered as variable modifications. For identification of farnesylation and geranylgeranylation sites, farnesylation (+161.18199 Da) and geranylgeranylation (+229.24458 Da) of protein C-terminal cysteine were set as variable modifications and digest specificity was set to semi-specific, considering that these modifications occur followed by proteolytic cleavage and C-terminal capping by methylation (2). The following parameters were applied: specific or semi-specific strict trypsin (trypsin/P), precursor and fragment mass tolerance of 20 ppm with mass calibration and parameter optimization enabled, up to two missed cleavages, and minimal peptide length of 6 amino acids. Match between runs was enabled in the mouse organ profiling experiment. Other parameters remained at the default settings. False discovery rates were estimated by searching against a reversed decoy database and filtered for <1% at the peptide-spectrum match (PSM) level.

In the case of HeLa cell profiling (related to Fig. 3), MaxQuant (v.2.1.1.0) (14) was utilized, since MaxQuant allows simultaneous analysis of peptides prepared by different digestive enzymes. The parameters described above were employed, and the other parameters remained at the default settings. False discovery rates (FDR) were estimated by searching against a reversed decoy database and filtered for <1% at the PSM level. Peptides with posterior error probability <1 were accepted.

For confident identification of lipidated peptides in addition to FDR control, only those that met at least one of the following criteria were accepted in the solvent comparison dataset (Fig. 2), HeLa cell dataset (Fig. 3), and mouse organ dataset (Fig. 4): (1) the lipidated site is reported in the UniProt database, (2) the site was identified in two or more unique peptides, (3) the site was indicated by a peptide identified from three or more MS/MS spectra in which three or more sequential b- or y-ions were observed. In the case of farnesylation and geranylgeranylation sites, in addition to the above criteria, only sites on the protein C-terminus with a C*AAX* (*A*, aliphatic amino acid; *X*, any amino acid) motif (2) were accepted.

Raw files acquired in the DIA mode were processed using DIA-NN (v.1.8.1) (15) to perform library-free search against the UniProt/SwissProt mouse database described above with the following parameters: up to two missed cleavages, precursor charge state of 2 to 4, precursor *m/z* range of 500 to 740, fragment ion *m/z* range of 120 to 1800. The maximum number of variable modifications was set to 3, and protein N-terminal methionine excision, cysteine carbamidomethylation, methionine oxidation, and acetylation on protein N-terminus were considered. Match-between-runs were enabled.

Supplemental Tables S1 and S2 contain the raw intensities given by MaxQuant and MSFragger, respectively. In the quantitative analyses, raw intensities were log_2_-transformed and median-normalized. We have deposited all files generated in database searching together with the raw data to jPOST (16) (see the Data Availability section). MS/MS spectra of identified peptides can be seen by downloading and then loading the files in the viewers of MaxQuant (14, 17) or MSFragger (13).

### Downstream Analysis

A sequence logo was created using WebLogo (v.1.0) (18). Data visualization, Pearson correlation calculation, and hierarchical clustering were performed using the R framework (v.4.1.3) with the basic functions and the ggplot2 (v.3.3.6) and pheatmap (v.1.0.12) packages.

### Experimental Design and Statistical Rationale

**Fig. 1:**
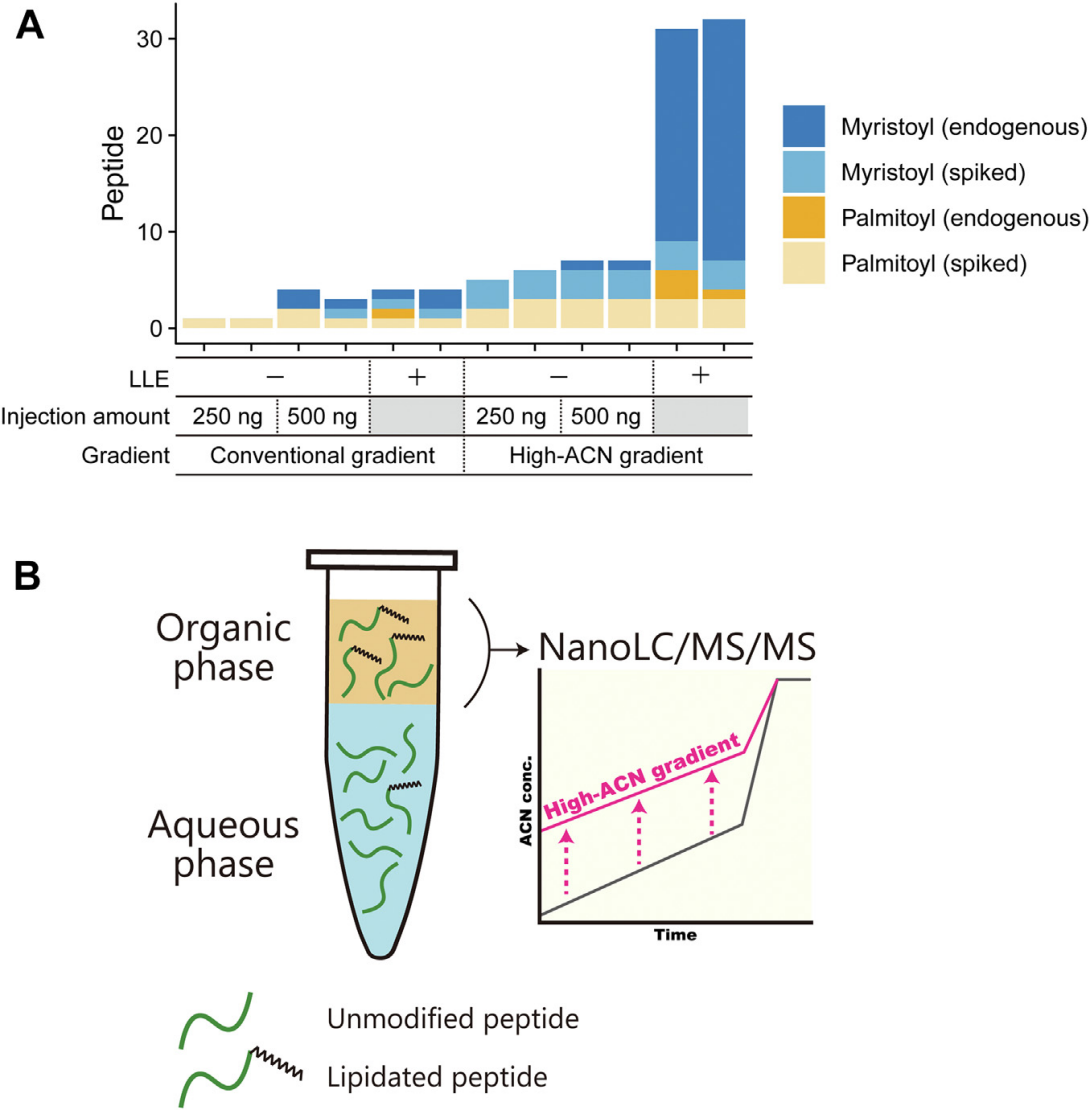
**Strategy for direct identification of lipidated peptides.***A*, comparison of the numbers of lipidated peptides identified by different methods. The numbers of identified myristoylated or palmitoylated peptides from HeLa cell protein digests spiked with synthetic lipidated peptides were compared with or without LLE using ethyl acetate and a conventional or high-ACN gradient. In the experiments with LLE used 25 μg peptides, while in the experiments without LLE, the amount of peptides injected was 250 ng or 500 ng, which is empirically considered to be approximately equal to the LLE-isolated peptides from 25 μg input. The spiked synthetic peptides are shown in supplemental Fig. S1. Conventional gradient: 4 to 36% acetonitrile (ACN) in 30 min, 36 to 80% ACN in 3 min, 80% ACN for 10 min. High-ACN gradient: 30 to 56% ACN in 30 min, 56 to 80% ACN in 3 min, 80% ACN for 10 min. *B*, scheme of extraction and analysis of lipidated peptides. Protein digests are prepared according to a conventional shotgun proteomics protocol. Hydrophobic lipidated peptides are partitioned into organic solvents and isolated by liquid-liquid extraction. Isolated peptides are analyzed by nanoLC/MS/MS, focusing on the high ACN concentration range.

**Fig. 2:**
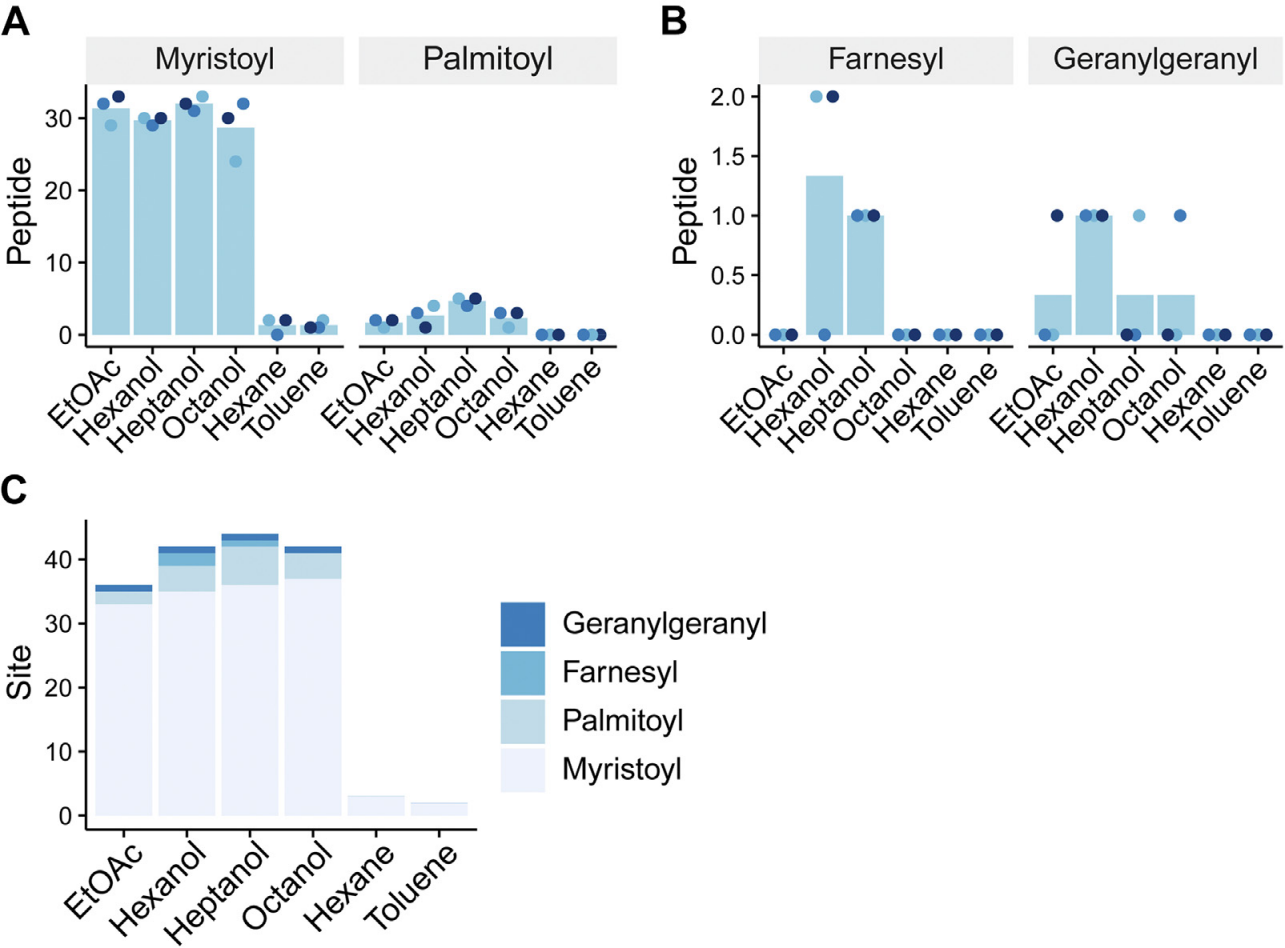
**Comparison of organic solvents for extraction of lipidated peptides.***A* and *B*, numbers of identified myristoylated peptides and palmitoylated peptides (*A*) and farnesylated peptides and geranylgeranylated peptides (*B*). The results of triplicate experiments are indicated in different colors. Bars indicate mean values. *C*, summary of the numbers of identified lipidation sites. The results of triplicate experiments are combined. EtOAc, ethyl acetate.

**Fig. 3:**
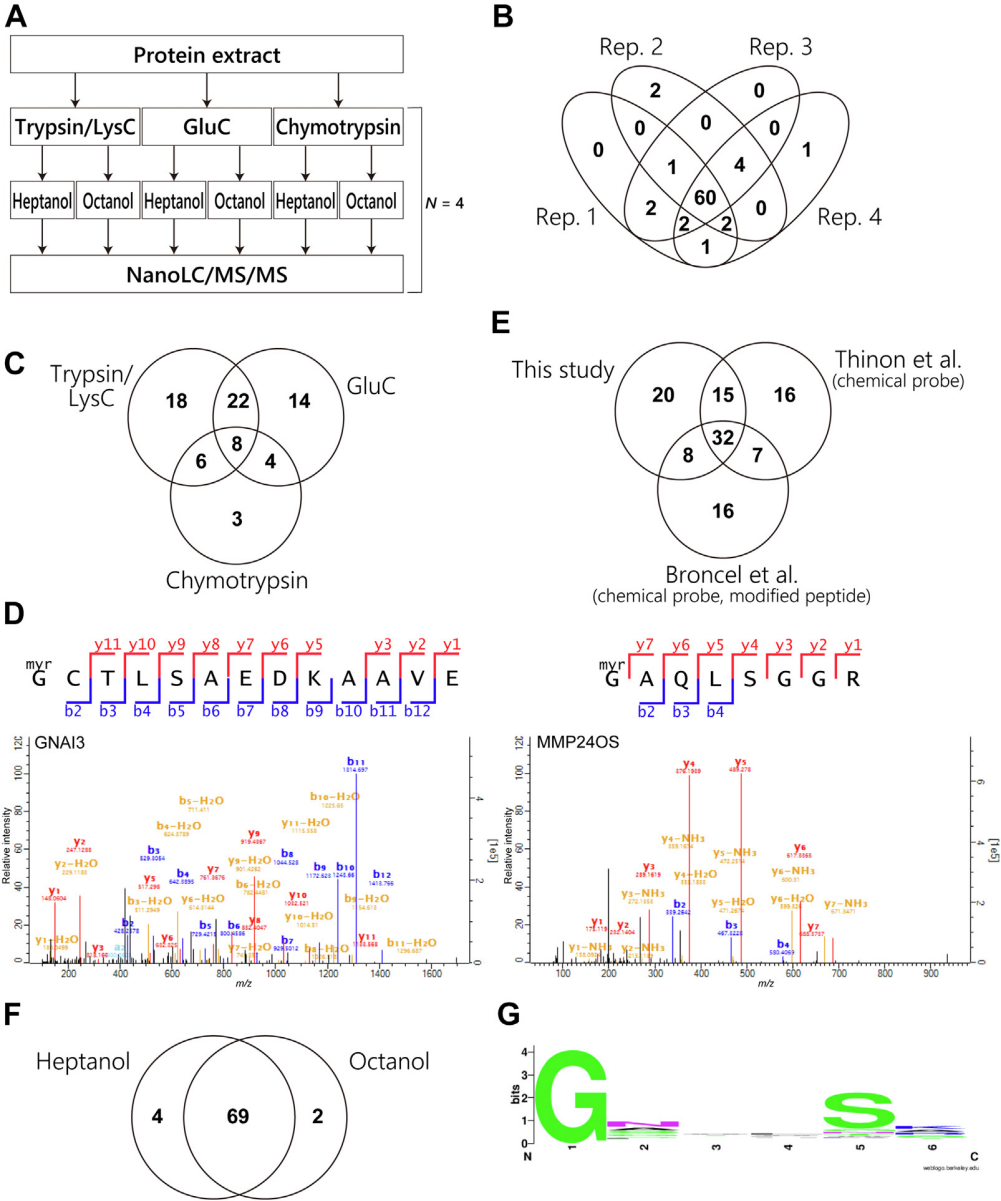
**Lipidation profiling of HeLa cell proteins.***A*, overview of the workflow. From the same HeLa cell extract, three digests were prepared using different proteases. From each digest, lipidated peptides were isolated using heptanol or octanol in parallel and analyzed by nanoLC/MS/MS in quadruplicate. *B*, overlap of identified myristoylation sites among the technical quadruplicates. *C*, overlap of identified myristoylation sites among the digestive enzymes. *D*, selected MS/MS spectra of identified myristoylated peptides. *Left*, GNAI3. *Right*, MMP24OS. *E*, comparison of identified myristoylated proteins with the reported studies. *F*, overlap of identified myristoylation sites between two organic solvents utilized for LLE, heptanol and octanol. *G*, sequence logo showing amino acids downstream of the identified protein N-terminal myristoylation sites.

**Fig. 4:**
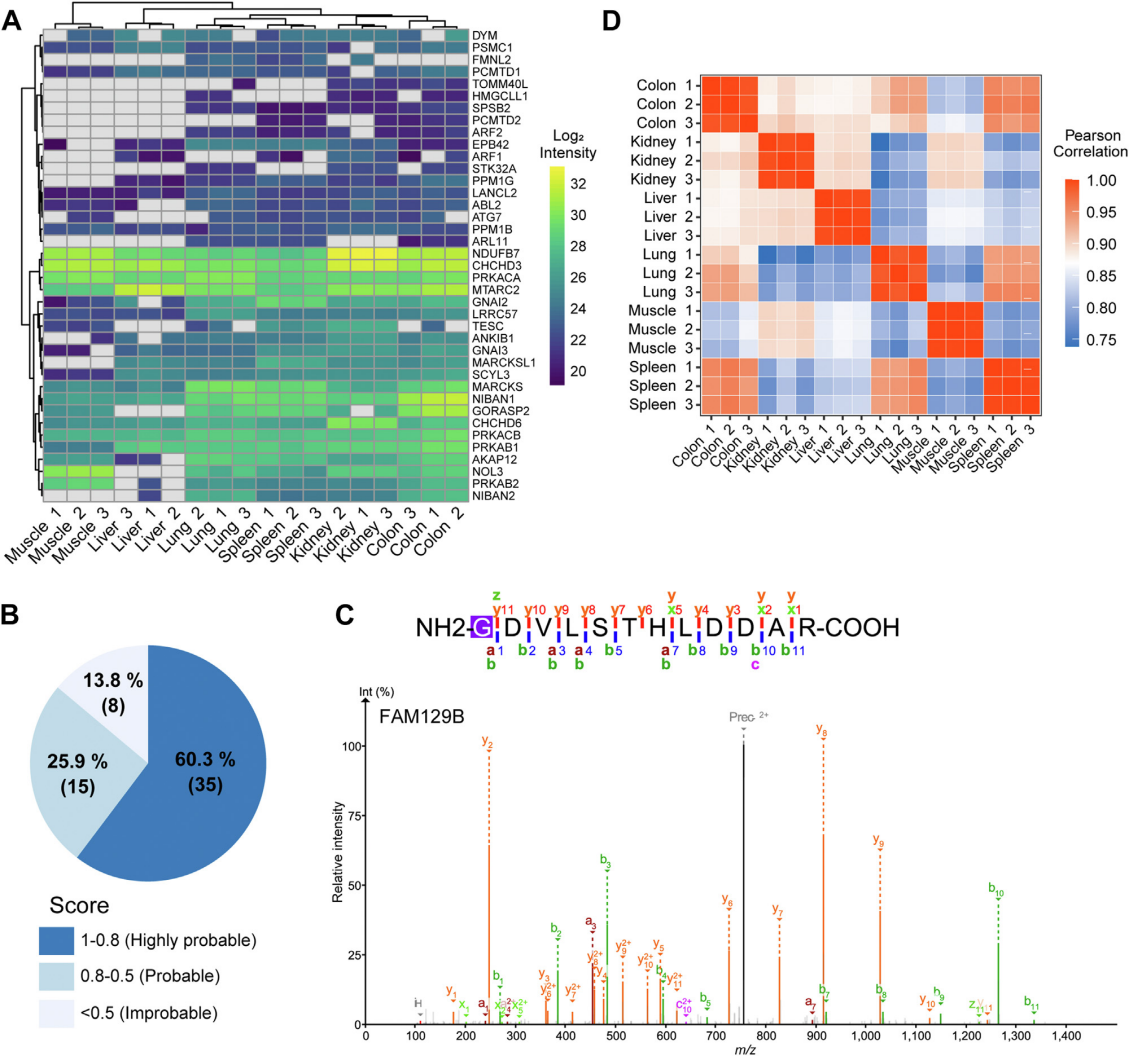
**Comparison of myristoylation sites among mouse tissues.***A*, myristoylation sites in six mouse tissues are compared in technical triplicates. Note that the sites were filtered to missing value <20% for clustering, reducing the number of sites to 39. *B*, SVMyr score proportion of the identified myristoylated proteins. *C*, MS/MS spectrum of the FAM129B myristoylated peptide. *D*, Pearson correlation coefficients of the intensities.

For each experiment, multiple LLE experiments were conducted using the same protein digests, which are referred to as experimental replicates. In the solvent comparison experiments (Fig. 2), three technical replicates of LLE were prepared, and each replicate was measured by 4 LC/MS/MS runs with varying FAIMS CVs as described in the nanoLC/MS/MS section above. In the experiments using synthetic peptides, peptides were analyzed by single nanoLC/MS/MS without FAIMS (supplemental Fig. S1 and Fig. 1*A*). For HeLa cell extracts (Fig. 3*A*), protein digests were prepared with trypsin/LysC, GluC, and chymotrypsin, in parallel. Then, for each digest, LLE was performed with heptanol and octanol, in parallel. For each solvent, three technical replicates were prepared, and each was measured by 4 LC/MS/MS runs with varying FAIMS CVs. Mouse organ proteins were digested with trypsin/LysC (Fig. 4). Then, three technical replicates of LLE were conducted with hexanol, and each was measured in 4 LC/MS/MS runs with varying FAIMS CVs. Mouse organ whole proteomes were quantified by technical duplicates in the DIA mode with fixed FAIMS CV. The number of replicates prepared is also indicated in the figure legends. No statistical analysis was performed in this study.

## Results and Discussion

### Isolation of Lipidated Peptides by Liquid-Liquid Extraction

For the detection of lipidated peptides by nanoLC/MS/MS, we first evaluated the chromatographic behavior of six synthetic lipidated peptides (three N-terminally myristoylated peptides and three cysteine *S*-palmitoylated peptides) spiked into HeLa tryptic digests. As expected, the lipidated peptides were eluted in a higher ACN concentration range (>32% ACN) than many other peptides (supplemental Fig. S1), indicating that a special gradient condition is needed for analysis of hydrophobic lipidated peptides, as previously suggested by other groups (8, 9). Using these synthetic peptides spiked into HeLa digests, we confirmed that coupling LLE and a high-ACN gradient provided much better identification efficiency of lipidated peptides than employing either alone (Fig. 1*A*). We, therefore, sought to analyze lipidated peptides more effectively by using the strategy shown in Figure 1*B*. First, similar to the conventional shotgun proteomics workflow, proteins extracted from cells are reduced, alkylated, digested, and desalted. Secondly, highly hydrophobic lipidated peptides from the protein digest are separated using LLE, where lipidated peptides partition into low-polarity organic solvents. Thirdly, we employ nanoLC/MS/MS with an atypical gradient program that starts from 30% ACN for the detection of lipidated peptides.

We first examined if peptides with N-terminal myristoylation at glycine or palmitoylation at cysteine residues are enriched using LLE. Previous studies by the Jensen’s group have shown that lipidated peptides can be isolated from protein digests by LLE (10, 11), but only ethyl acetate was tested as an organic solvent. We thus optimized LLE with various organic solvents, namely ethyl acetate, hexanol, heptanol, octanol, hexane, and toluene using a trypsin/LysC digest of 50 μg of extracted HeLa cell proteins. More than 30 myristoylated peptides and several palmitoylated peptides were extracted by ethyl acetate, hexanol, heptanol, or octanol (Fig. 2*A*). On the other hand, only a few myristoylated or palmitoylated peptides were extracted by hexane and toluene. We also examined if peptides with C-terminal farnesylation or geranylgeranylation at a cysteine residue can be enriched by an additional semi-specific search, as these modifications occur followed by proteolytic cleavage (2). As a result, one or two farnesylated or geranylgeranylated peptides were identified using hexanol, heptanol, and octanol, but no sites were identified using hexane or toluene (Fig. 2*B*).

Compared to myristoylation, fewer sites were found for other lipidations, including palmitoylation. We assume that one of the reasons for this is the low stoichiometry of the modified forms. Co-translational protein N-terminal myristoylation would proceed at a high stoichiometric level during translation and is irreversible, whereas cysteine palmitoylation is dynamic through the interplay between palmitoyl acyltransferases and palmitoyl-protein thioesterase (19) and probably exists at a low stoichiometric level under basal conditions. This possibility is supported by the fact that a recently published method for enriching *S*-acylated peptides by solid-phase extraction requires as much as 7.5 mg of peptides as input (20), which is 150-fold higher input than this experiment. Note that the partition efficiency of palmitoylated peptides is similar to or higher than that of myristoylated peptides (supplemental Fig. S2). Also, prenylation is known to accelerate the degradation of some substrates (21, 22), which would make them more difficult to detect. Taken together, we concluded that LLE is especially suitable for the extraction of myristoylated peptides.

### Myristoylation Profiling of HeLa Cells

As a proof-of-concept of this methodology, we performed a comprehensive profiling of protein N-terminal myristoylation sites in HeLa cells. To expand the coverage, proteins were digested with trypsin/LysC, GluC, or chymotrypsin, in parallel (Fig. 3*A*). Aliquots of 100 μg of digests were subjected to LLE, in which heptanol and octanol were employed; thus, a total of 600 μg peptides was used per replicate. The myristoylation sites were reproducibly identified in the technical quadruplicates (Fig. 3*B*). Using trypsin/LysC, 54 myristoylation sites were identified (Fig. 3*C*), which is similar to the number of myristoylation sites identified by Castrec *et al.* (8) utilizing a combination of subcellular fractionation, biochemical peptide separation, and nanoLC/MS/MS. To extend the sequence coverage, the use of multiple digestive enzymes in parallel is effective (23). It is noteworthy that combined use of multiple digestive enzymes increased the number of identified sites (Fig. 3*C*). The use of GluC and chymotrypsin enabled detection of 18 and 7 myristoylation sites that were not identified as tryptic peptides, respectively (Fig. 3*C*). We identified a total of 75 myristoylation sites (supplemental Table S1 and Fig. 3*D*), which is greater than the number of the high-confidence myristoylated proteins in one of the largest current myristoylome datasets, constructed by Thinon *et al.* (5) (Fig. 3*E*). Broncel *et al.* (6) developed a multifunctional chemical probe that enables identification of myristoylation sites. Compared to the results of using that probe for HeLa cells, our method yielded 40 sites that overlapped, but identified more sites in total (Fig. 3*E*). We utilized two solvents for LLE, but most of the identified peptides were overlapped (Fig. 3*F*), suggesting that the use of multiple digestive enzymes, rather than multiple solvents, is more effective to expand coverage of *N*-myristoylome.

The sequences flanking modified glycine in our dataset are shown as a sequence logo in Figure 3*G*, in which serine at the +5 position is highlighted. This motif is often observed in the case of protein N-terminal glycine myristoylation (24), supporting the validity of our dataset. The identified sites included many known modification sites based on the report by Meinnel (25). We also found three sites not reported, including dynamin-2 (DNM2), guanine nucleotide-binding protein G(s) subunit alpha isoforms short (GNAS), MMP24OS (Fig. 3*D*, right), indicating the ability of our methodology to identify unknown lipidated proteins at the site level. Interestingly, we detected three peptides dually modified with myristoylation and palmitoylation, derived from tyrosine-protein kinase YES1 (supplemental Fig. S3), guanine nucleotide-binding protein G(i) subunit alpha-3 (GNAI3), and raftlin. Notably, the dually modified peptide of YES1 was uniquely identified by chymotrypsin digestion because of a peptide of sufficient length for mass spectrometric detection, again highlighting the importance of using multiple enzymes. Simultaneous observation of such multiple modifications is a major advantage of our methodology since it is difficult to obtain information about modified sites by chemical proteomic approaches using lipid-mimetic probes.

### Identification of *In Vivo* Myristoylation Sites in Mouse Tissues

Finally, we demonstrate that our methodology enables quantitative analysis of *in vivo* myristoylation sites in mouse tissues. We prepared protein digests from six mouse tissues (kidney, muscle, colon, spleen, lung, and liver) using trypsin, and myristoylated peptides were isolated using heptanol in three technical replicates. We chose the combination of trypsin for digestion and heptanol for LLE because this combination yielded the best result (Figs. 2*C* and 3C). A total of 58 myristoylation sites were identified (Fig. 4*A* and supplemental Fig. S5; supplemental Table S2). FAIMS CV −60 V gave the largest number of myristoylated peptides (supplemental Fig. S4). Among them, 23 sites, such as NIBAN1 and ABL2, were identified in all organs, while eight sites, such as formin-like protein 3 (FMNL3) and HID1, were uniquely identified in a single organ (supplemental Fig. S5). Muscle presented the lowest number of myristoylation sites (22 sites), while colon presented the largest number (47 sites). Among the myristoylated proteins not detected in muscle, 12 were not expressed at the protein level (supplemental Fig. S6), accounting in part for the lower number of identified myristoylation sites in muscle. In contrast, interestingly, despite the ubiquitous expression of BLOC-1-related complex subunit 5 (BORCS5), its myristoylation was specifically observed only in the spleen (supplemental Fig. S5). We compared the results with those generated by a recently released web server for myristoylation prediction, SVMyr (26). Most (86.2%) of the identified myristoylated proteins were annotated as highly probable or probable, whereas 8 (13.8%) were annotated as improbable (Fig. 4*B*). However, we found that the proteins annotated as improbable include a myristoylated protein identified with high confidence based on the MS/MS spectrum (Fig. 4*C*), underscoring the strength of direct identification of myristoylation sites using our method, as compared with computational prediction. The samples were segregated based on organs by hierarchical clustering (Fig. 4*A*). The Pearson correlation coefficients of log intensities of myristoylated peptides between the technical replicates in each organ were more than 0.98, whereas those between different tissues were lower (Fig. 4*D*).

Taken together, our results indicate that this methodology is applicable for the identification of *in vivo* modification sites and is sufficiently reproducible to permit differential analysis.

## Conclusion

We have demonstrated the utility of LLE for N-terminal myristoylation proteomics. Our method does not require the cells to incorporate exogenous chemical probes and is therefore applicable to intact biological samples. Furthermore, using alternative proteases such as GluC and chymotrypsin extended the coverage of myristoylation sites. Application of our approach to mouse tissues revealed tissue-specific myristoylation, providing a target for future biological study. Very recently, Ji *et al.* (20) showed that nano graphite fluoride-based solid-phase extraction is effective in enriching cysteine *S*-acylated peptides, but not *N*-myristoylated peptides. Therefore, our method and the solid-phase extraction by Ji *et al.* (20) can be considered as complementary techniques for capturing different types of lipidation. This also indicates that further development is required to simultaneously capture a variety of lipid modifications. In summary, this simple and rapid method should be useful to investigate biological machinery regulated by lipidation of functional proteins, particularly the role of myristoylation.

## Data Availability

The proteomics data have been deposited to the ProteomeXchange Consortium *via* the jPOST partner repository (16) with the dataset identifier PXD041499 (JPST002094).

## Supplemental Data

This article contains supplemental data.

## Conflict of interest

The authors declare that they have no conflicts of interest with the contents of this article.

## Supplemental Data

Supplemental Figures S1–S6

Supplemental Table S1 List of identified myristoylated peptides and sites in HeLa cells.

Supplemental Table S2 List of identified myristoylated sites in mouse organs.
