# Supplementary material for: Application of Liquid-Liquid Extraction for N-terminal Myristoylation Proteomics
Source: Mol Cell Proteomics. 2023 Nov 9;22(12):100677. doi: 10.1016/j.mcpro.2023.100677 (PMC10696250; doi:10.1016/j.mcpro.2023.100677)
Supplement: Supplemental Figures S1–S6 [file mmc1.docx]

**Application of liquid-liquid extraction for N-terminal myristoylation proteomics**

Kazuya Tsumagari^1,2,3^, Yosuke Isobe^2,4,7^, Yasushi Ishihama^5,6^, Jun Seita^3^, Makoto Arita^2,4,7,8*^, and Koshi Imami^1,2,3,^*

^1^Proteome Homeostasis Research Unit, RIKEN Center for Integrative Medical Sciences, Tsurumi-ku, Yokohama, Kanagawa 230-0045, Japan.

^2^Laboratory for Metabolomics, RIKEN Center for Integrative Medical Sciences, Tsurumi-ku, Yokohama, Kanagawa 230-0045, Japan.

^3^Laboratory for Integrative Genomics, RIKEN Center for Integrative Medical Sciences, Tsurumi-ku, Yokohama, Kanagawa 230-0045, Japan.

^4^Division of Physiological Chemistry and Metabolism, Graduate School of Pharmaceutical Sciences, Keio University, Minato-ku, Tokyo 105-8512, Japan.

^5^Department of Molecular Systems Bioanalysis, Graduate School of Pharmaceutical Sciences, Kyoto University, Kyoto 606-8501, Japan.

^6^Laboratory of Clinical and Analytical Chemistry, National Institute of Biomedical Innovation, Health and Nutrition, Ibaraki, Osaka 567-0085, Japan.

^7^Cellular and Molecular Epigenetics Laboratory, Graduate School of Medical Life Science, Yokohama City University, Tsurumi-ku, Yokohama, Kanagawa 230-0045, Japan.

^8^Human Biology-Microbiome-Quantum Research Center (WPI-Bio2Q), Keio University, Tokyo, Japan.

 * **For correspondence**: koshi.imami@riken.jp (KI), makoto.arita@riken.jp (MA)


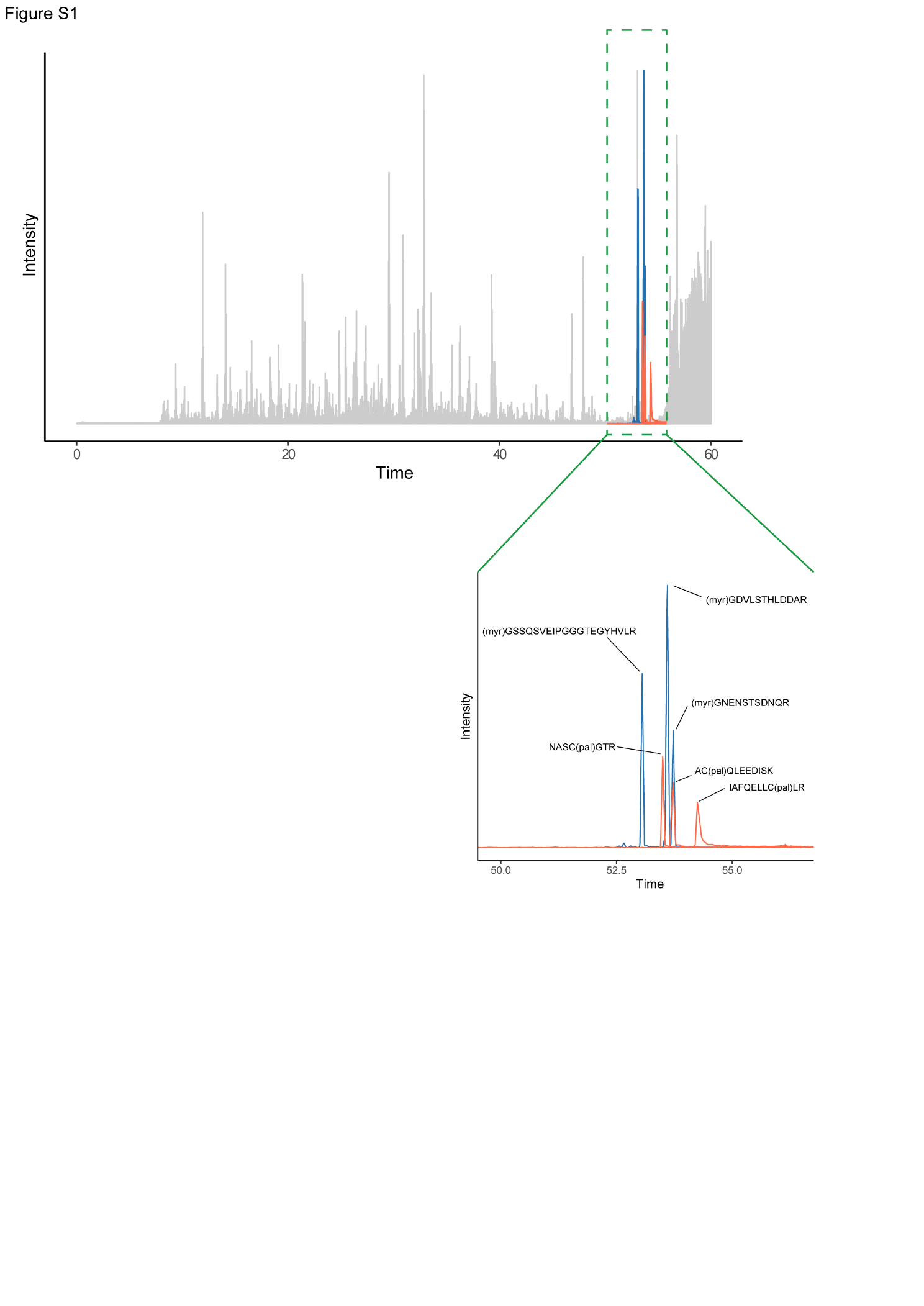


**Figure S1. Comparison of retention times of HeLa protein digest and synthetic lipidated peptides.**

Synthetic myristoylated peptides and palmitoylated peptides were spiked into HeLa protein digests and analyzed by nanoLC/MS/MS. The extracted ion chromatograms of myristoylated peptides (blue) and palmitoylated peptides (orange) are overlayed on the base-peak chromatogram. Note that intensities are normalized for ease of visualization.

**
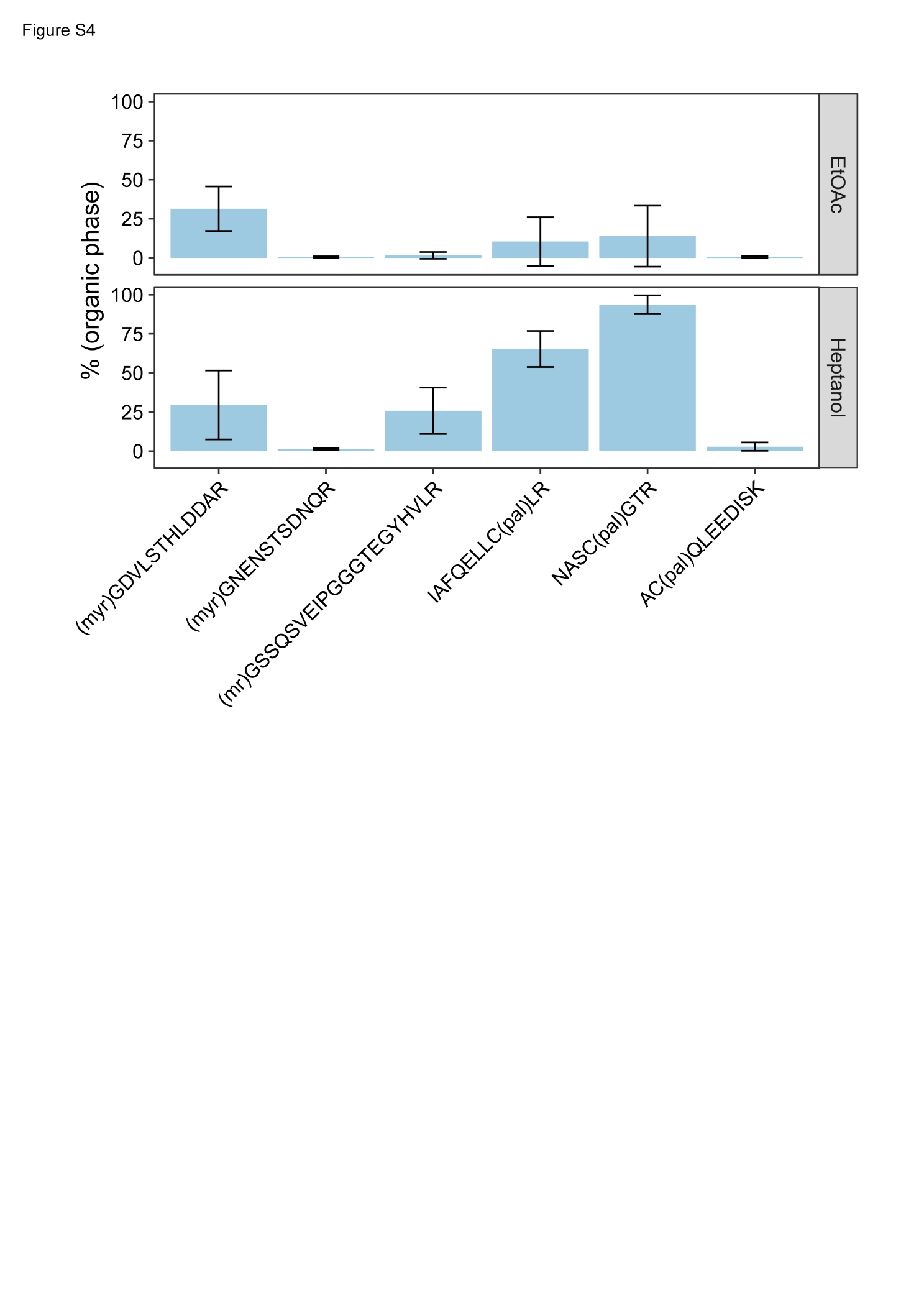
**

**Figure S2. Partition efficiency of lipidated peptides.**

The percentage of peptides that partitioned into the organic phase was determined by dividing the intensity of peptides in the organic phase by the sum of the intensity of peptides in the organic and aqueous phases. Synthetic lipidated peptides were utilized. The experiments were done in triplicate, and the data are presented as mean ± standard deviations.


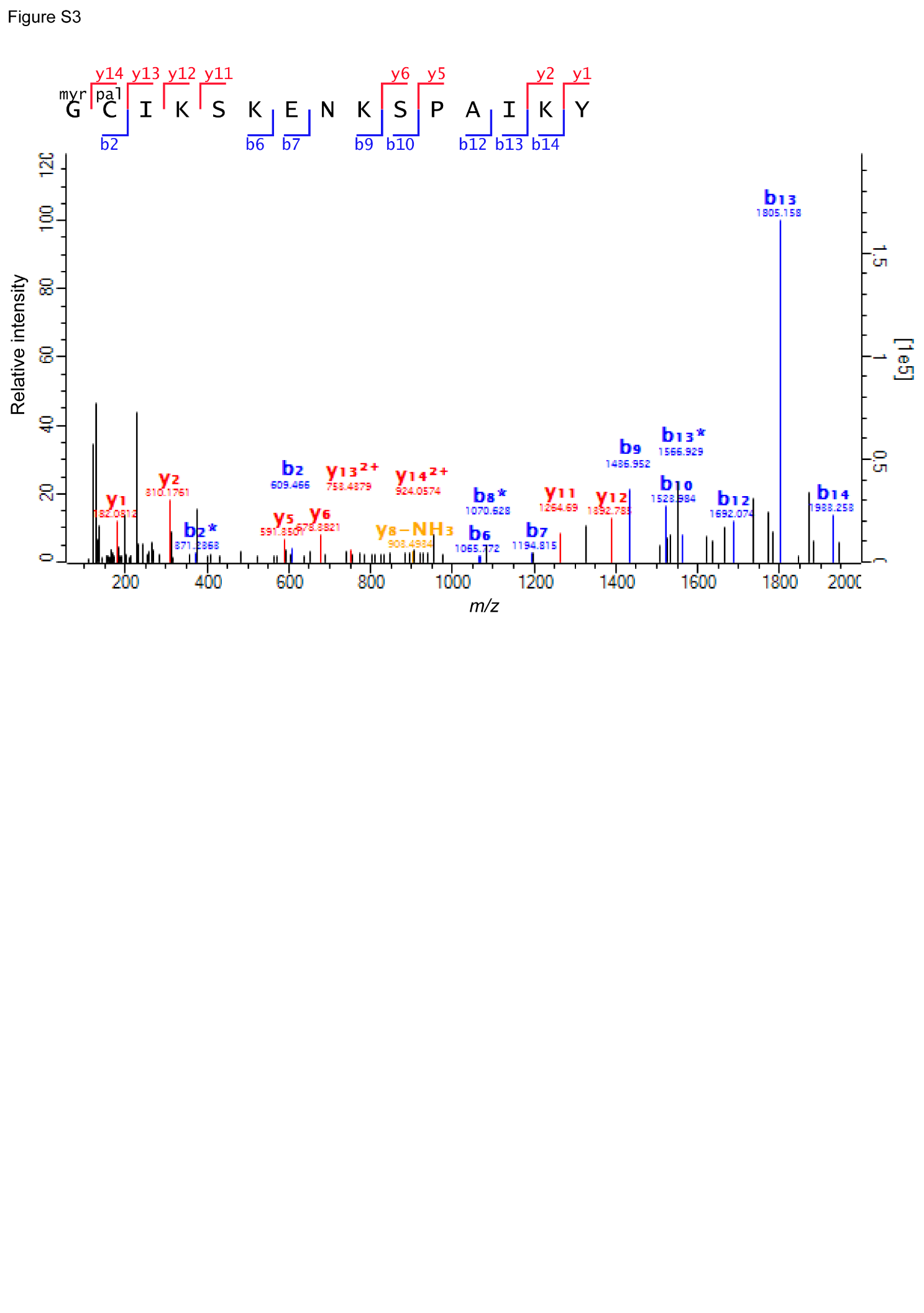


**Figure S3. MS/MS spectrum of dually modified YES1 peptide in HeLa cells.**


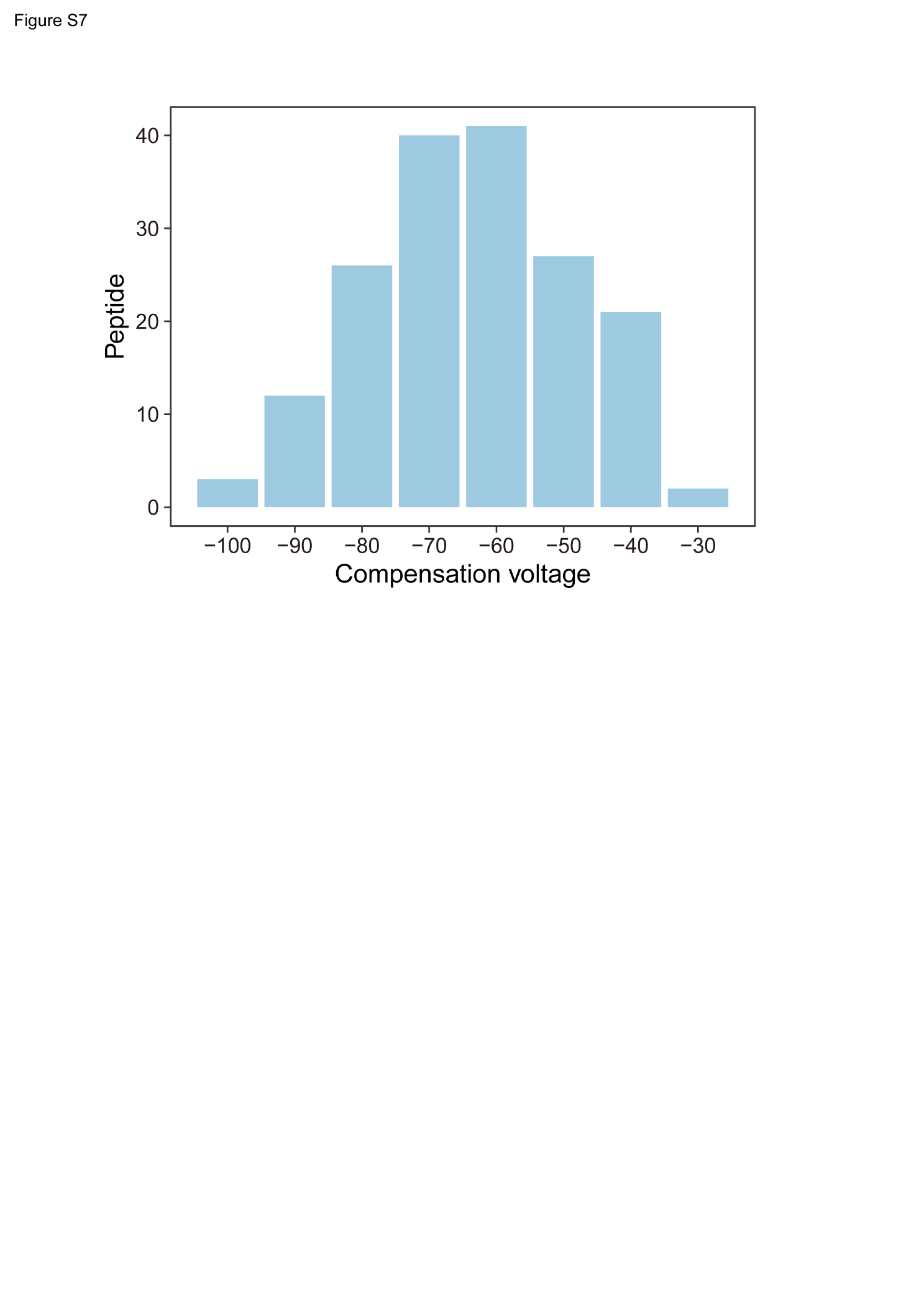


**Figure S4. Number of identified peptides using different FAIMS CVs.**

The number of identified unique peptides from mouse organs in the measurements with each compensation voltage of FAIMSpro.


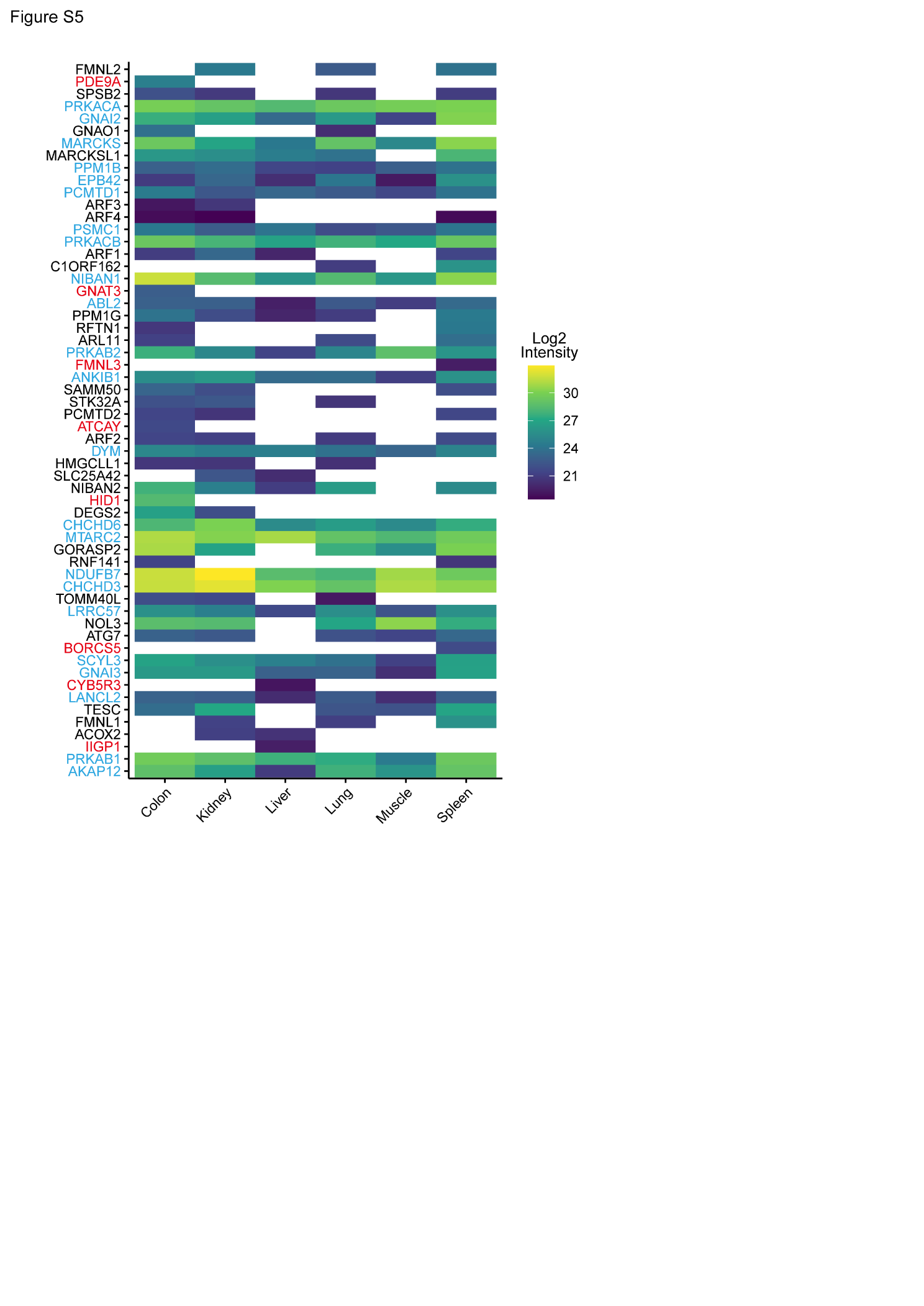


**Figure S5. Protein N-terminal myristoylation sites detected in mouse organs.**

Myristoylation sites identified in six mouse organs. Mean values of technical triplicates are shown. The sites uniquely identified in one organ and the sites commonly identified in all organs are highlighted in red and blue, respectively.


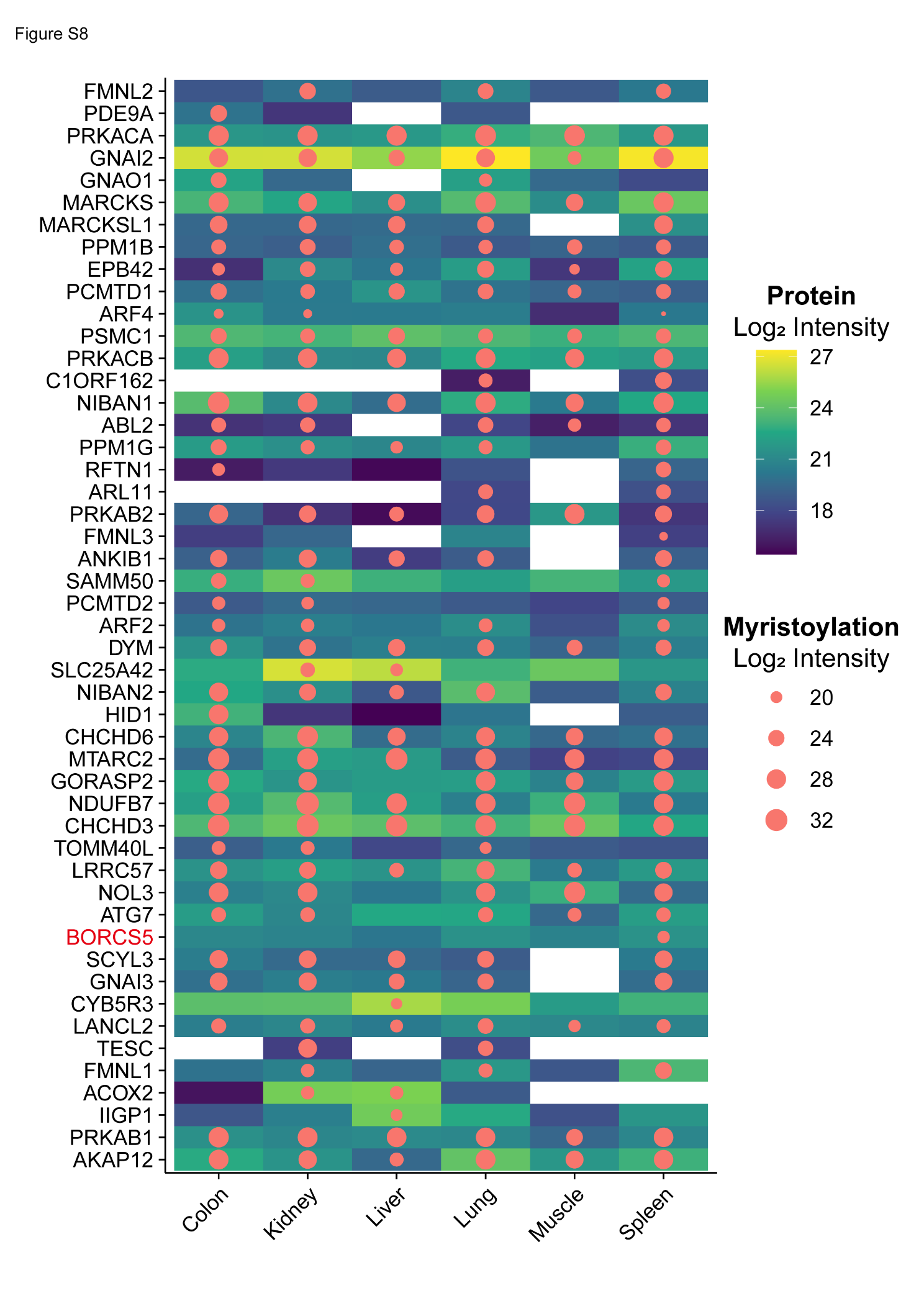


**Figure S6. Expression levels of the identified myristoylated proteins in mouse organs.**

Protein expression levels of the identified myristoylated proteins shown as a heatmap. The circles reflect their myristoylation levels (Fig. S5).
